# Predictors of Worse Prognosis in Young and Middle-Aged Adults Hospitalized with COVID-19 Pneumonia: A Multi-Center Italian Study (COVID-UNDER50)

**DOI:** 10.3390/jcm10061218

**Published:** 2021-03-15

**Authors:** Martina Bonifazi, Federico Mei, Edlira Skrami, Lara Letizia Latini, Donatella Amico, Elisabetta Balestro, Francesco Bini, Floriano Bonifazi, Antonella Caminati, Piero Candoli, Saverio Cinti, Susanna Contucci, Alessandro Di Marco Berardino, Sergio Harari, Guido Levi, Sara Lococo, Vincenzo Menditto, Giampietro Marchetti, Sara Piciucchi, Venerino Poletti, Claudia Ravaglia, Marina Saetta, Gianluca Svegliati-Baroni, Sara Tomassetti, Mario Tamburrini, Alessandro Zanforlin, Umberto Zuccon, Lina Zuccatosta, Stefano Gasparini, Flavia Carle

**Affiliations:** 1 Department of Biomedical Sciences and Public Health, Marche Polytechnic University, 60020 Ancona, Italy; letizia.latini@outlook.it (L.L.L.); s.gasparini@univpm.it (S.G.); 2 Respiratory Diseases Unit, Azienda Ospedaliero-Universitaria “Ospedali Riuniti”, 60126 Ancona, Italy; fedusmei@gmail.com (F.M.); horatio1983@libero.it (A.D.M.B.); linazuccatosta@tiscali.it (L.Z.); 3 Center of Epidemiology, Biostatistics and Information Technology, Department of Biomedical Sciences and Public Health, Marche Polytechnic University, 60020 Ancona, Italy; f.carle@univpm.it; 4 Respiratory Diseases Unit, “Ospedali Riuniti Marche Nord”, 61032 Pesaro, Italy; donatella.amico@ospedalimarchenord.it (D.A.); piero.candoli@ospedalimarchenord.it (P.C.); 5 Department of Cardio-Thoracic-Vascular Sciences and Public Health, University of Padova, 35127 Padova, Italy; elisabetta_balestro@hotmail.com (E.B.); saralococo.sl@gmail.com (S.L.); marina.saetta@unipd.it (M.S.); 6 Respiratory Diseases Unit, “Guido Salvini” Hospital, 20021 Garbagnate Milanese, Italy; francesco_bini@hotmail.com; 7 Health Department “Regione Marche”, 60125 Ancona, Italy; floriano.bonifazi@hotmail.com; 8 Division of Pulmonary and Critical Care Medicine, San Giuseppe Hospital MultiMedica IRCCS, 20123 Milan, Italy; antonella.caminati@multimedica.it; 9 Department of Experimental and Clinical Medicine, Center of Obesity, Marche Polytechnic University, 60020 Ancona, Italy; s.cinti@univpm.it; 10 Emergency Unit, Azienda Ospedaliero-Universitaria “Ospedali Riuniti”, 60126 Ancona, Italy; susanna.contucci@ospedaliriuniti.marche.it (S.C.); vincenzogiannicola.menditto@ospedaliriuniti.marche.it (V.M.); 11 Department of Clinical Sciences and Community Health, University of Milan, 20123 Milan, Italy; sergio@sergioharari.it; 12 Department of Medicine, San Giuseppe Hospital MultiMedica IRCCS, 20123 Milan, Italy; 13 Respiratory Diseases Unit, “Spedali Civili” Hospital, 25123 Brescia, Italy; guido.levi@yahoo.it (G.L.); marchetti.giampietro@libero.it (G.M.); 14 Radiology Department, “Giovan Battista Morgagni e Luigi Pierantoni” Hospital, 47121 Forli, Italy; piciucchi.sara@gmail.com; 15 Respiratory Diseases Unit, Department of Diseases of the Thorax, Ospedale GB Morgagni, 47121 Forli, Italy; venerino.poletti@gmail.com (V.P.); claudiaravaglia79@gmail.com (C.R.); 16 Department of Respiratory Diseases and Allergy, Aarhus University Hospital, DK-8200 Aarhus, Denmark; 17 Liver Diseases and Transplant Unit, Azienda Ospedaliero-Universitaria “Ospedali Riuniti”, 60126 Ancona, Italy; g.svegliati@univpm.it; 18 Obesity Center, Marche Polytechnic University, 60020 Ancona, Italy; 19 Department of Experimental and Clinical Medicine, “Careggi” Hospital, 50134 Florence, Italy; s.tomassetti@gmail.com; 20 Respiratory Diseases Unit, “Santa Maria degli Angeli” Hospital, 33170 Pordenone, Italy; mario.tamburrini@asfo.sanita.fvg.it (M.T.); umberto.zuccon@asfo.sanita.fvg.it (U.Z.); 21 Pneumology Service, Azienda Sanitaria dell’Alto Adige, 39100 Bolzano, Italy; alessandro.zanforlin@gmail.com

**Keywords:** obesity, asthma, comorbidity, mechanical ventilation, pneumonia, COVID-19, young people, mortality, predictors

## Abstract

Obesity as well as metabolic and cardiovascular comorbidities are established, significant predictors of worse prognosis in the overall COVID-19 population, but limited information is available on their roles in young and middle-aged adults (aged ≤ 50 years). The main objectives of the present Italian multi-center study were to describe clinical characteristics and role of selected prognostic predictors in a large cohort of young and middle-aged hospitalized patients. Nine pulmonology units, across north and center of Italy, were involved in this retrospective study. Comorbidities were classified according to their known or potential association with COVID-19. A total of 263 subjects were included. The prevalence of obesity was 25.9%, mechanical ventilation (MV) was needed in 27.7%, and 28 in-hospital deaths occurred (10.6%). Obesity and older age were the only independent, significant predictors for MV. Comorbidities, such as hypertension, diabetes, asthma, and increased D-dimer levels were significantly associated with higher mortality risk, regardless of age, body mass index, and MV. Obesity in young and middle-aged adults is a strong predictor of a more complicated COVID-19, without, however, evidence of a significant effect on in-hospital mortality. Selected comorbidities, including hypertension, diabetes and asthma, significantly impact survival even in a younger population, suggesting the need for prompt recognition of these conditions.

## 1. Introduction

In December 2019, an outbreak of novel coronavirus disease (COVID-19), occurred in Wuhan, a city in the Chinese province of Hubei, and, thereafter, it has dramatically spread worldwide [1,2]. The COVID-19 caused by SARS-CoV-2 can manifest with mild or no symptoms or can lead to serious and fatal respiratory illness and/or multiorgan failures, and predictors of worse prognosis include older age, male gender, and pre-existing comorbidities [3]. The majority of patients who develop a moderate to severe disease are actually aged more than 50 years old, but a significant minority of younger patients requiring hospitalization and mechanical ventilation has been also observed, with an overall case-fatality rate of less than 1% [4]. Moreover, in Italy, over the recent months, the median age of diagnosis has lowered to 44 years, with almost the half of affected subjects aged less than 50 years [4]. However, limited information on clinical characteristics, outcomes and relative predictors in this subgroup are currently available. In particular, obesity seems to be a significant risk factor for a more severe disease [5], but its specific burden in young people has yet to be fully addressed. Obese subjects, indeed, are usually prone to develop complicated respiratory diseases [6] due to the variable interplay between altered mechanical dynamics in ventilation and chronic inflammatory/immune dysregulation [7]. Again, the relevant role of selected comorbidities, such as diabetes and cardiovascular diseases, in increasing the risk of a worse prognosis in the overall population has been extensively assessed worldwide [3], but a specific focus in young and middle-aged patients is currently lacking.

Therefore, the main objectives of the present Italian multi-center study were to describe the clinical characteristics, and the role of selected predictors of worse prognosis, in particular obesity, in a large cohort of hospitalized patients with SARS-CoV2 pneumonia aged less than or equal to 50 years.

## 2. Materials and Methods

Nine pulmonology units, over the north and center of Italy, were involved in this multi-center observational study. Anonymous data of patients included in the study cohort were retrospectively collected from electronic medical records. The study protocol was approved by the coordinator ethics committee (n. 2020131, date of approval 7 May 2020) and by each local ethics committee and the need for patient’s informed consent was waived.

### 2.1. Patients and Clinical Information

Consecutive patients, aged 18–50 years, hospitalized for confirmed or probable diagnosis of SARS-CoV2 infection between 28 February and 15 May, were included in the present study. A confirmed case was defined as a person with laboratory confirmation of COVID-19, while a probable case as a person with suggestive clinical/radiological features with an inconclusive/negative laboratory test or for whom testing could not be performed for any reason [8].

Demographic factors and selected clinical characteristics were collected for all cases. These included: age, sex, smoking history, height, weight, comorbidities, gas exchange values (P/F [pO2/FiO_2_]), extent of lung involvement at chest X-ray (CRX), parenchymal pattern at high resolution computed tomography (HRCT), d-dimer value, interleukin 6 (IL-6), creatin kinase value, alanine aminotransferase (ALT), need for oxygen support, cycles of pronation. The follow-up period was the time from admission to the date of discharge or death. Cycles of high flow nasal cannula oxygen (HFNC) and/or non-invasive mechanical ventilation (nIMV)—indicated with SpO_2_ < 92% on oxygen therapy 15 l/min FiO_2_ 50%), the need for mechanical ventilation (IMV)—indicated when the respiratory rate was above 25/min and/or signs of acute respiratory failure, despite HFNC/nIMV, days from hospitalization to mechanical ventilation, days from hospitalization discharge, main clinical outcomes (death, discharge), were collected during the follow-up period.

Body mass index (BMI) was calculated as weight (kg)/height (m)^2^ and categorized into three levels: up to normal weight (BMI < 25 kg/m^2^), overweight (25 ≤ BMI < 30 kg/m^2^) and obese (BMI ≥ 30 kg/m^2^).

Mechanical ventilation (MV) was classified as: no MV, nIMV (for subjects requiring only nIMV), and invasive (for patients requiring only IMV or nIMV followed by IMV).

Comorbidities were classified according to their known or potential association with COVID-19, as reported in the literature [2,3,9,10]. Comorbidity was defined as a “high risk for COVID-19” when at least one of the following conditions was present: hypertension, coronary heart disease, chronic heart failure, stroke, COPD, asthma, interstitial lung disease, diabetes, immunosuppression, oncologic diseases and chronic kidney disease; comorbidity was defined as “other”, in case of no evidence nor rationale for association with COVID-19, and “absent”, when none of the above mentioned conditions were present.

The baseline CXR of each patient included was evaluated by the local radiologist to assess the extent of lung involvement by means of a semi-quantitative score, adapted from the Radiographic Assessment of Lung Edema score [11]. Each lung was assessed individually, and depending on the extent of consolidation or ground-glass opacity, a score of 0 to 4 points was given (0—no involvement; 1—less than 25%; 2—25% to 50%; 3—50% to 75%; 4—more than 75% involvement). The overall score was the sum of points from both lungs. Lung involvement was also classified as mild (total score ≤ 2) and moderate-severe (total score > 2).

When available, HRTCs were also evaluated in terms of qualitative radiological pattern.

### 2.2. Statistical Analysis

The normality of the distribution of the variables was evaluated by means of the Shapiro test. The 95% confidence intervals (95% CI) for the proportion of IMV and nIMV were estimated using binomial distribution.

Missing data were calculated for each variable and available case analysis was used in order to avoid further decrease in the size of the dataset. Quantitative variables were summarized using median and interquartile range (1–3 quartiles), and qualitative variables as absolute and percentage frequencies. The characteristics of the study sample were described stratifying for MV. A chi-squared for trend test (or Fisher exact test) was used to evaluate linear trends in the distribution of categorical variables through MV categories; Kruskal–Wallis test was performed to compare groups in case of quantitative variables.

In multiple models, variables statistically significant at the descriptive analysis and/or those with clinical relevance were considered as explanatory variables; MV was dichotomized as the need for mechanical ventilation (invasive or non-invasive) and no need for mechanical ventilation, due to the small number of patients requiring invasive MV.

A multiple logistic regression analysis was performed to evaluate the role of clinical characteristics and BMI on the probability to require MV. The model was adjusted for age, gender, smoke status and comorbidities. The likelihood ratio (LR) and Hosmer-Lemeshow (HL) tests were used to select the most parsimonious model and to evaluate the model’s goodness of fit. Generalized variance-inflation factors was used for the collinearity diagnostics in all multiple regression models.

The Kaplan Meyer method was used to estimate the survival probability in hospital. Comparison between survival curves stratified for MV were performed using the Log-rank test.

A weighted Cox regression analysis was used to estimate the effect of clinical characteristics of patients on the probability of death, adjusted for age and comorbidities. The model goodness of fit was evaluated using the Wald chi-square test. The determinant of the covariance matrix of the weighted Cox regression estimates was used for the collinearity diagnostics.

All the analyses were performed using R statistical package 4.0.2 [12] and statistical significance was assessed using a level of probability of 5%.

## 3. Results

### 3.1. Patients Characteristics

A total of 268 subjects were included in the present study and in 263 of them information on the study outcomes were available. Demographic and clinical characteristics at baseline (i.e., on hospital admission), stratified for the need of mechanical ventilation, are summarized in Table 1, while missing variables with relative percentages are shown in Table 2.

All patients, except one, received a confirmed diagnosis of SARS-CoV2 infection. Overall, nIMV was needed for 40 patients (17.9%; 95% CI: 13.1%; 23.6%), whereas IMV was needed in 33 patients (14.5%; 95% CI: 10.1%; 19.6%). The median age on admission was 45 years (1; 3rd quartiles: 40.4; 48.4), the majority of patients were males and non-smokers, without significant differences according to subgroups. The median level of BMI was 26 kg/m^2^ (1; 3 quartiles: 23.3; 30.0), and the proportion of obese patients (BMI ≥ 30 kg/m^2^) was significantly higher in subjects who received mechanical ventilation compared to those who did not. No comorbidities were reported for approximately half of patients, one-quarter of the cohort presented with conditions known or suspected to be associated with a higher risk of infection, and the remaining subjects had other pathological entities. Hypertension, diabetes and asthma were the most prevalent conditions, reported respectively in 35 (14.8%), 18 (6.8%), and 15 subjects (5.7%); these conditions also represented the 72% of the high-risk comorbidities group. Three out of 15 asthmatic subjects also had hypertension (*n* = 2), diabetes (*n* = 1), and four had a BMI ≥ 30 kg/m^2^. No significant differences in comorbidities prevalence were observed according to need for mechanical ventilation or not.

Patients requiring MV were significantly older, had significantly higher levels of IL-6, worse P/F ratios at baseline, showed a greater lung impairment (score > 2), and received more frequent pronation cycles, as compared to those not requiring MV. Patients requiring IMV were characterized by significantly worse P/F ratios, higher levels of D-dimer and IL-6, a higher lung involvement (score > 2), and received more frequent pronation cycles and HFNC, compared to patients with no need for MV. Significantly higher levels of D-dimer and a higher frequency of pronation were also found in patients requiring IMV compared to those requiring nIMV. Patients did not differ in terms of gender, smoking habits, comorbidities, HRCT pattern, CPK and ALT levels. The median time of follow-up was 8 days (1; 3 quartiles: 4; 11 days) in patients that did not require MV, 12 days (1; 3 quartiles: 8; 19 days) in patients requiring non-invasive MV and 21 days (1; 3 quartiles: 15; 31 days) in patients requiring invasive MV.

### 3.2. Outcomes

Table 3 shows the results of the multiple logistic regression analysis. The need for MV was significantly associated with BMI and age. In particular, the risk of receiving MV in obese patients was almost 3.5-fold higher compared to patients with normal BMI (<25 kg/m^2^); regarding age, the probability of requiring mechanical ventilation increased about 7% for every added year of age. The same factors were found also to be significantly associated with the probability of requiring nIMV compared to the need of no MV.

During the follow-up period, a total of 28 deaths were observed. The survival probability at 38 days from hospitalization was 58.6% (95%CI: 38.1–90.0%) in patients not requiring MV, 70.1% (95% CI: 49.7%; 98.9%) in patients requiring nIMV and 67.2% (95%CI: 46.0%; 98.1%) in those requiring invasive MV, and no statistically significant differences were observed.

Patients with “high risk” comorbidities had a risk of death 6.51 times higher than those without comorbidities (95% CI: 2.07–20.48; *p* = 0.001) (Table 4); a significant excess risk was found also in patients with high D-dimer levels (HR 1.28, 95% CI: 1.04 1.57; *p* = 0.017) and the probability of death increased about 28% for every added unit (mg/mL) (Table 4).

## 4. Discussion

The present study firstly provides a detailed assessment of the clinical impact of obesity and further selected predictors of worse prognosis in a large cohort of young and middle-aged patients hospitalized with COVID-19, derived from nine pulmonology units across the north and the center of Italy. Overall, obesity and older age were the only significant predictors for MV (either invasive or non-invasive) in a full model adjusted for comorbidities and markers of severity, like D-dimer levels. In particular, the prevalence of subjects with a BMI ≥ 30 kg/m^2^ was over 40% in subgroups receiving mechanical ventilation and the relative risk in these patients was more than three-fold higher compared to patients with normal BMI. Further relevant findings are that short-term in-hospital mortality rate in young and middle-aged people was not negligible and that independent predictors of worse prognosis were comorbidities, and higher D-dimer levels on admission. Notably, the six-times mortality risk excess associated with “high risk” comorbidities was due to the most prevalent conditions, hypertension, diabetes and asthma, regardless of age, BMI, and having received MV or not.

The prevalence of obesity in our hospitalized cohort (25.9%) was higher than that reported by age class in Italy (around 6% in subjects aged 18–45 years in 2019) [13], suggesting that this might be a risk factor for both hospitalization and a more severe disease in the young also. Our results are in line with previous observations in the adult COVID-19 population, as summarized by meta-analyses of data, showing increased risks of hospitalization, severe disease, the need for MV and mortality in obese patients, with different levels of risk excess [14,15,16,17]. However, in our cohort, obesity, the heaviest independent determinant for MV, was not associated with a higher mortality risk. One possible explanation is related to the age class we focused on. It is reasonable to speculate that systemic and mechanical alterations in obese subjects made the need for an intensive ventilatory support more likely, without, however, significantly influencing survival (if appropriately ventilated) in the absence of further significant comorbidities (less likely in younger subjects). Two recent studies have also focused on young and middle-aged adults (<50 years) [18,19], exploring predictors for hospital admission, clinical characteristics and management. Both these studies have confirmed the significant association between obesity and a more severe COVID-19, but no data were reported on its impact on mortality. Thus, our study is the first one providing information on role of obesity in influencing survival in young and middle-aged populations. Therefore, the apparent discrepancy of our finding with the findings reported by Hoong et al. [17] could be due to the older age of the patients included in the studies considered in the review.

A strong association between obesity and complicated viral infections has been already documented for H1N1 influenza A [6], as well as for the previous coronaviruses causing widespread infections (SARS, MERS) [5]. Adipocytes play a relevant role in several physiologic and metabolic processes and an excess in adipose tissue leads to increased levels of adipokines and inflammatory cytokines [20]. Such subtle, chronic inflammation is thought to cause immune dysfunction, impairing the adaptive immune response to viral infections and favoring the development of immune-mediated diseases. One of the mechanisms leading to worse outcomes in obese COVID-19 patients includes a dysfunction of specific fat-resident regulatory T cells (Treg) and TH17 (T-cell sub-lineage), as a result of high circulating IL-6, IL-23/IL-17, as well as other obesity-associated plasma cytokines, contributing to the ‘cytokine storm’ described in more serious cases [5]. In our study, the median IL-6 level in subjects with BMI ≥ 30 kg/m^2^ (30.6 pg/mL) was, indeed, two-times higher compared to those with lower BMIs (15.5 pg/mL), although the difference was not significant, probably due to the high variability of IL-6 levels. Moreover, a greater susceptibility to virus permeability in the airways has been suggested in obese subjects, as adipocytes express the membrane-bound glycoprotein angiotensin-converting enzyme 2 (ACE2), known to be the entry point of the virus, and, thus, an excess in adipose tissue is believed to increase the viral up-take.

A further potential mechanism in increasing the risk of more severe diseases in obesity may be related to hypercoagulation status. Notably, coagulation and fibrinolysis are altered in obesity, mainly due to high plasma concentrations of multiple prothrombotic factors [21]. Moreover, according to a new elegant hypothesis, SARS-CoV-2 fat infiltration could worsen the adipose inflammatory status and infected adipocytes would massively undergo necrotic death, exacerbating lipid remnant accumulation in the adipose interstitium, with free lipid droplets that, in turn, could predispose to fat embolism syndrome [21]. In fact, fat embolism was documented in autoptic lung specimens from two adult COVID-19 patients with overweight [21], suggesting that this might be a possible pathological event that needs further investigation.

Furthermore, the potential role of unbalanced dietary intake in obesity, poor in vitamins and antioxidants, is likely to contribute to immune dysfunction. Severe vitamin D deficiency, commonly observed in COVID-19 hospitalized patients [22] and in obese subjects [23] may directly promote hypertension and impact on the renin–angiotensin system components that could contribute to target organ damage.

Lastly, altered mechanical dynamics in obese subjects might favor complicated respiratory infections, due to a preexisting functionally restrictive pattern, as well as a tendency to airway hyperresponsiveness and earlier bronchiolar closure [7,15].

Another interesting finding is that comorbidities not particularly severe, such as hypertension and asthma, were significant, independent, strong predictors of in-hospital mortality even in a young population. Whereas the relevant role of hypertension in increasing the likelihood of a more severe diseases has been fully established [3], evidence on chronic obstructive respiratory diseases, in particular on asthma, are less robust. A meta-analysis of literature did not show a significant relationship between asthma and COVID-19 [24], but a slightly higher mortality rate in asthmatic subjects using high dose inhaled corticosteroids or the combination with long-acting beta-agonist or oral corticosteroids, deemed as surrogates of a more severe disease [3]. In our study, asthma was the third most prevalent comorbidity, and results from Cox regression analysis suggest that it might play a role in increasing mortality risk in young patients, although its specific burden was not estimated due to the small number of observations (15 patients and 4 deaths). However, the lack of information on asthma severity and pheno-endotypes in our cohort makes difficult to properly interpret this finding. Patients suffering from different asthma pheno-endotypes are likely to present with different risk profiles in terms of infection, and progression to severe COVID-19 outcomes [25,26]. In particular, Zhu et al. explored the association between asthma and severe COVID-19 according to the phenotypes “allergic asthma” and “non-allergic asthma” and found a significantly higher risk in the latter group only [21]. The inflammatory response in non-type 2 asthma is dominated by type 1 and/or type 17 T-cell and many of these asthmatics suffer from comorbidities, including obesity, glucose dysregulation and hypertension as part of the metabolic syndrome, characterized by higher plasma levels of IL-6 [27].

The major strengths of this study include the relatively large number of observations, considering the low prevalence of young and middle-aged adults hospitalized for COVID-19 in the first pandemic wave, and the representativeness of the study population, as different centers across Italy were involved. Furthermore, our study is the first study to investigate the independent role of obesity on poor prognosis and on mortality separately in COVID-19 patients less than or equal to 50 years old. Interesting information on the effects of comorbidities on COVID-19 prognosis in young and middle-aged adults are also provided.

The study has some limitations. Due to the retrospective data collection, there are a number of missing collected characteristics, as they were not reported in medical records and it was not possible to achieve patients at home after discharge. Therefore, it was not possible to evaluate the role of some clinical predictors, as IL-6 levels and instrumental severity indicators (radiological score, tomography) on worse prognosis and mortality; moreover, the sample size in the multiple analysis was reduced respecting the number of the recruited patients.

Despite the relatively large number of observations, the studied sample size was not sufficient to estimate the role of single comorbidities, such as asthma, on COVID-19 prognosis.

Data included in this study were collected during the first dramatic wave of the pandemic and limited to fundamental clinical information; thus we did not have the opportunity of looking at genetic predispositions for severe COVID-19 and for metabolic disorders in young people.

Lastly, pressure on the health system and the different internal guidelines adopted by each hospital involved in this study might have influenced the occurrence of outcomes, such indication to MV, in the different Italian areas included in this study.

## 5. Conclusions

In conclusion, our findings suggest that obesity in young and middle-aged adults is a strong, independent, predictor of a more complicated COVID-19 infection, without, however, evidence for a significant effect on in-hospital mortality. On the other hand, selected comorbidities, including hypertension, diabetes and asthma, significantly impacted survival even in a young adult population.

In light of the increasing global prevalence of obesity, hypertension and asthma, any effort should be made to further promote a healthier lifestyle through educational programs, and implement strategies to drastically reduce factors that contribute to chronic respiratory conditions, such as air pollution.

Due to the significant prognostic implications, a prompt recognition of these comorbidities in young COVID-19 patients is highly recommended and closer surveillance of this subgroup is warranted. Further studies are needed to better define pathogenetic mechanisms and potential targeted management.

## Figures and Tables

**Table 1:** Distribution of patients’ demographic and clinical characteristics according to the need of mechanical ventilation.

	Mechanical Ventilation				*p* *
	Total	No	nIMV	IMV	
Variables	*n* = 263	*n* = 190	*n* = 40	*n* = 33	
Gender, *n* (%)					
Males	164 (62.4)	115 (60.5)	28 (70.0)	21 (63.6)	0.801
Females	99 (37.6)	75 (39.5)	12 (30.0)	12 (36.4)	
Age, years [median (1; 3 quartiles)]	45.3 (40.4; 48.4)	43 (34.0; 47.8)	46 (42.0; 49.0)	45 (40.0; 47.0)	0.004 ^#^
Groups compared with a statistically significant difference		no vs. nIMV			
Smoke, *n* (%)					
Never	145 (68.4)	103 (66.9)	22 (66.7)	20 (80.0)	0.695
Current/Former	67 (31.6)	51 (33.1)	11 (33.3)	5 (20.0)	
BMI classes, *n* (%)					
<25 kg/m^2^	87 (44.2)	70 (51.5)	9 (23.7)	8 (34.8)	0.012
25–29 kg/m^2^	59 (29.9)	41 (30.1)	13 (34.2)	5 (21.7)	0.627
≥30 kg/m^2^	51 (25.9)	25 (18.4)	16 (42.1)	10 (43.5)	0.001
Comorbidity, *n* (%)					
No	130 (51.2)	94 (50.5)	22 (56.4)	14 (48.3)	0.982
Other	58 (22.8)	46 (24.7)	5 (12.8)	7 (24.1)	0.811
“high risk for COVID-19”	66 (26.0)	46 (24.7)	12 (30.8)	8 (27.6)	0.794
P/F ratio [median (1; 3 quartiles)]	299 (219.0; 383.5)	360.5 (295.0; 402.5)	218.5 (156.2; 273.3)	199.9 (158.6; 263.0)	<0.001 ^#^
Groups compared with a statistically significant difference		no vs. nIMV		no vs. nIMV	
RX lung involvement, *n* (%)					
mild (score ≤ 2)	45 (42.9)	43 (54.4)	1 (6.7)	1 (9.1)	<0.001 ^§^
moderate-severe (score > 2)	60 (57.1)	36 (45.6)	14 (93.3)	10 (90.9)	
Groups compared with a statistically significant difference		no vs. nIMV		no vs. nIMV	
HRCT pattern, *n* (%)					0.236 ^§^
pure GGO	23 (22.8)	10 (15.9)	9 (47.4)	4 (21.1)	
crazy paving	9 (8.9)	7 (11.1)	1 (5.3)	1 (5.3)	
GGO with consolidations	59 (58.4)	39 (61.9)	8 (42.1)	12 (63.2)	
consolidations	10 (9.9)	7 (11.1)	1 (5.3)	2 (10.5)	
D-dimer, µg/mL [median (1; 3 quartiles)]	0.4 (0.3; 0.8)	0.4 (0.3; 0.7)	0.5 (0.2; 0.9)	0.9 (0.5; 1.6)	0.001 ^#^
Groups compared with a statistically significant difference			nIMV vs. IMV	no vs. nIMV	
IL-6, pg/mL [median (1; 3 quartiles)]	28.7 (8.1; 50.2)	9 (3.8; 23.8)	24.3 (8.7; 49.9)	49 (12.8; 77.3)	<0.001 ^#^
Groups compared with a statistically significant difference		no vs. nIMV		no vs. nIMV	
CPK, U/l [median (1; 3 quartiles)]	76 (44.0; 142.0)	69 (43.0; 121.0)	100 (58.0; 206.0)	76 (41.0; 234.5)	0.249 ^#^
ALT, U/l [median (1; 3 quartiles)]	34 (22.0; 58.0)	33 (21.0; 59.0)	35 (27.5; 70.0)	42.5 (21.5; 63.0)	0.278 ^#^
O2-therapy, *n* (%)					0.017
No	88 (34.9)	88 (48.6)	0 (0)	0 (0)	
Yes	164 (65.1)	93 (51.4)	40 (100)	31 (100)	
Pronation, *n* (%)					<0.001 ^§^
No	217 (91.9)	174 (98.9)	32 (91.4)	11 (44.0)	
Yes	19 (8.1)	2 (1.1)	3 (8.6)	14 (56.0)	
Groups compared with a statistically significant difference		no vs. nIMV	nIMV vs. IMV	no vs. nIMV	
HFNC, *n* (%)					0.004 ^§^
No	86 (92.5)	73 (97.3)	9 (81.8)	4 (57.1)	
Yes	7 (7.5)	2 (2.7)	2 (18.2)	3 (42.9)	
Groups compared with a statistically significant difference				no vs. nIMV	

* Chi-squared for trend test; ^#^ Kruskal-Wallis test; ^§^ Fisher exact test; ALT: alanine aminotransferase; BMI: body mass index; CPK: creatin kinase; GGO: ground glass opacity; HFNC: high flow nasal cannula; HRCT: high resolution computed tomography; IL-6: interleukin-6; IMV: invasive mechanical ventilation; nIMV: non-invasive mechanical ventilation; P/F: pO2/FiO_2_.

**Table 2:** Missing data distribution for the variables evaluated in the study (*n* = 268 subjects included in the study).

Variables	Missing, *n* (%)
Mechanical Ventilation	5 (1.9)
Death	0 (0)
Gender	0 (0)
Age	0 (0)
Smoke	52 (19.4)
BMI	66 (24.6)
Hypertension	1 (0.4)
ACE	1 (0.4)
CHD	1 (0.4)
CHF	1 (0.4)
COPD	1 (0.4)
Asthma	1 (0.4)
Interstitial lung disease	1 (0.4)
Diabetes	1 (0.4)
Other comorbidities	13 (4.9)
P/F ratio	55 (20.5)
Rx lung involvement	158 (59)
HRCT pattern	165 (61.6)
D-dimer	55 (20.5)
IL-6	136 (50.7)
CPK	64 (23.9)
ALT	5 (1.9)
02-therapy	14 (5.2)
Pronation	31 (11.6)
HFNC	173 (64.6)

Footnotes. ALT: alanine aminotransferase; ACE: angiotensin-converting enzyme; BMI: body mass index; CHD: cardiovascular heart disease; CHF: cardiovascular heart failure; COPD: chronic obstructive pulmonary disease; CPK: creatin kinase; GGO: ground glass opacity; HFNC: high flow nasal cannula; HRCT: high resolution computed tomography; IL-6: interleukin-6; IMV: invasive mechanical ventilation; nIMV: non-invasive mechanical ventilation; P/F: pO2/FiO_2_.

**Table 3:** Factors associated with the need for mechanical ventilation (IMV or nIMV vs. no need). Results of the multiple logistic regression analysis.

Variable	OR	95% CI	*p*
BMI, 25–29 kg/m^2^ vs. < 25 kg/m^2^	1.43	0.54; 3.81	0.467
BMI, ≥ 30 kg/m^2^ vs. < 25 kg/m^2^	3.50	1.44; 8.79	0.006
D-dimer, µg/mL	0.91	0.66; 1.19	0.530
Comorbidity, Other vs. No	0.85	0.29; 2.38	0.756
Comorbidity, “high risk” vs. No	1.06	0.43; 2.63	0.899
Death, Yes vs. No	2.00	0.67; 6.06	0.213
Age, years	1.07	1.01; 1.14	0.021
Gender, Female vs. Male	0.68	0.29; 1.55	0.363
Smoke, Yes vs. No	0.59	0.24; 1.42	0.251

The model was based on 139 observations; D-dimer and age were included as continuous variables. LR test: *p* = 0.028; HL test: *p* = 0.783; GVIF (all) < 1.3; BMI: body mass index; OR: odds ratio; 95% CI: 95% confidence interval.

**Table 4:** Factors associated with death. Results of the Cox regression analysis.

Variable	HR	95% CI	*p*
BMI, 25–29 kg/m^2^ vs. > 25 kg/m^2^	0.29	0.05; 1.74	0.175
BMI, ≥ 30 kg/m^2^ vs. < 25 kg/m^2^	0.79	0.27; 2.27	0.655
Comorbidity, Other vs. No	2.23	0.33; 15.03	0.410
Comorbidity, “high risk” vs. No	6.51	2.07; 20.48	0.001
Mechanical ventilation, Yes vs. No	0.82	0.30; 2.18	0.684
D-dimer, µg/mL	1.28	1.04; 1.57	0.017
Age, years	0.93	0.87; 0.98	0.012

The model was based on 153 observations; D-dimer and age were included as continuous variables; Wald Chi-square test: *p* = 0.001; determinant of covariance matrix = 5.10 × 10^−7^; BMI: body mass index; HR: hazard ratio; 95% CI: 95% confidence interval.

## Data Availability

The data presented in this study are available on request from the corresponding author. The data are not publicly available due to privacy policy of the centers involved in the study.
